# Anti-trapping scaffold protein: significance in cancer cell innate immune signaling and opportunities for immunotherapy

**DOI:** 10.3389/fimmu.2026.1905777

**Published:** 2026-09-16

**Authors:** Dawit Kidane

**Affiliations:** Department of Physiology and Biophysics, College of Medicine, Howard University, Washington, DC, United States

**Keywords:** cGAS-STING axis, genomic instability, innate immunity, PD-L 1, XRCC1

## Abstract

X-ray repair cross-complementing protein 1 (XRCC1) is a scaffold protein that functions as a molecular hub, serving as a platform for the recruitment and interaction of multiple DNA repair proteins to coordinate and facilitate multistep DNA repair processes. Furthermore, it acts as an antitrapping protein, ensuring the timely release of DNA repair proteins to prevent genotoxicity. XRCC1 participates in single-strand break repair (SSBR) pathways and has also been implicated in other DNA repair pathways. Preclinical studies have shown that XRCC1 deficiency delays SSB rejoining, induces mutations, and results in elevated levels of sister chromatid exchanges, a hallmark of genomic instability. XRCC1 deficiency results in hypersensitivity to ionizing radiation and chemotherapeutics. Analyses of the Cancer Genome Atlas (TCGA) datasets have revealed that approximately 30% of tumors exhibit low XRCC1 expression. Recently, Shahi et al. demonstrated that XRCC1 deficiency combined with pharmacological inhibition of Ataxia-Telangiectasia Mutated (ATM) induces mitotic failure, resulting in the accumulation of cytosolic DNA and activation of cancer cell-intrinsic innate immune signaling. Notably, this response was associated with increased PD-L1 expression, which may contribute to adaptive immune resistance. Collectively, these findings suggest that XRCC1 deficiency can promote cancer-cell-intrinsic molecular features associated with innate immune activation. This may represent a therapeutic vulnerability that warrants further evaluation in gastric cancer, particularly in studies combining DNA damage response (DDR) inhibition with immunotherapy.

## Introduction

Base excision repair (BER) is critical for the accurate removal of damaged DNA bases generated by oxidative and alkylating agents. DNA single-strand breaks (SSBs) induced by chemotherapeutics are processed by SSB repair (SSBR), which is closely related to BER. X-ray repair cross-complementing gene (XRCC1) is an essential BER factor that serves as a scaffold protein, coordinating the recruitment and interaction of multiple proteins at the site of DNA damage (1, 2). XRCC1 was the first mammalian gene identified to protect cells from the deleterious effects of ionizing radiation (3), and was subsequently shown to provide protection against multiple types of stress, including alkylation and UV-induced damage (4–6). XRCC1 lacks intrinsic enzymatic activity; instead, it functions as an interaction platform for multiple repair enzymes involved in the SSBR pathway (5, 7). Additionally, studies support the involvement of XRCC1 in other repair pathways, including the nonhomologous end-joining (NHEJ) pathway (8, 9). XRCC1 is a 70-kDa protein with three functional domains: an N-terminal DNA-binding domain, a centrally located BRCT Breast cancer gene 1 carboxyl-terminal domain (BRCT I) domain, and a C-terminal Breast cancer gene 2 carboxyl-terminal domain (BRCT II) domain, which are connected by two unstructured linker segments of approximately 150 and 120 residues. The XRCC1 N-terminal domain (X1NTD) binds specifically to DNA polymerase β (Polβ) (10, 11); the C-terminal BRCT domain (X1BRCTb) interacts with DNA ligaseIIIα (Lig3) (12). The central domain contains a poly(ADP-ribose) binding motif (13) that mediates XRCC1 recruitment to poly(ADP-ribose) generated following Poly(ADP-ribose) polymerase 1 (PARP1) activation at sites of single-strand breaks (14). Multiple studies have reported interactions between XRCC1 and DNA (15–19). Biochemical characterization showed that XRCC1 exhibits little affinity for double-stranded DNA (dsDNA), but relatively high affinity for nicked, gapped, or blunt-ended DNA. It has also been reported that the affinity of XRCC1 for DNA is inversely related to the degree of phosphorylation (15).

Furthermore, the Cancer Genome Atlas (TCGA) PanCancer datasets were analyzed for XRCC1 mRNA expression. Ten cancer types comprising 5,140 cases were included. In the gastric cancer cohort, approximately 30% of tumors exhibited low XRCC1 expression (**Figure 1A**). One potential mechanism underlying this reduced expression is hypermethylation of the XRCC1 promoter, which may suppress gene transcription and consequently decrease XRCC1 levels in cancer cells (20, 21). Additionally, a small-scale study using immunohistochemical analysis reported that approximately 77% of gastric cancer tissues exhibited low XRCC1 protein expression (22, 23). Moreover, reduced XRCC1 expression was associated with an increased incidence of precancerous lesions in several gastrointestinal organs, including the colon and liver, following exposure to alkylating agents (24), highlighting the biological and clinical significance of XRCC1 deficiency in gastric carcinogenesis.

**Figure 1:**
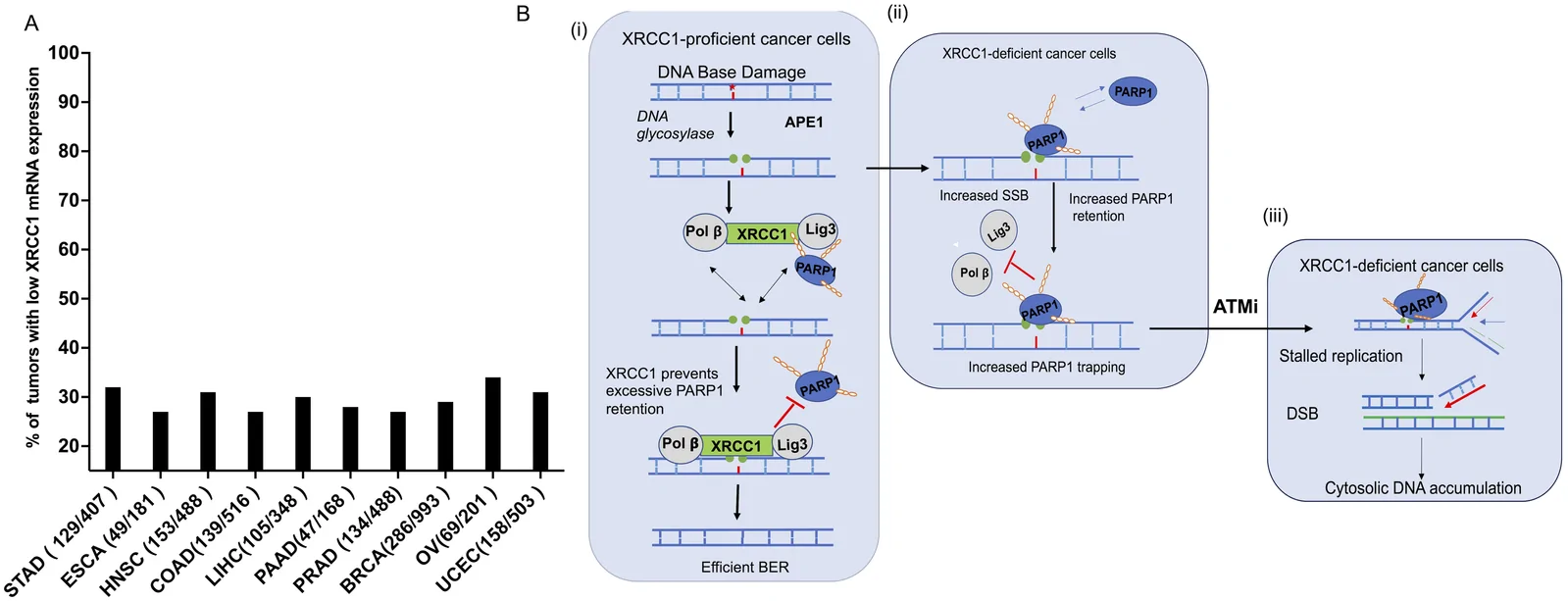
Low expression of XRCC1 in cancer and mechanism of repair defect. **(A)** Data were curated from TCGA PanCancer database accessed through cBioportal genomics, and XRCC1 mRNA *z*-score < -0.5 was classified as low expression. A total of 5,140 tumor cases from 10 cancer types with valid RNA-Seq V2 data were included. Patients were categorized into expression groups using *Z*-score values: those with *z*-score < -0.5 were classified as low expression, and those with *Z*-scores > 0.5 were categorized as high expression. Patients’ RNA-Seq expression *Z*-scores ranging between − 0.5 ≤ *Z* ≤ 0.5 were excluded from data analysis to enhance the contrast between high- and low-expression groups. All data were curated from TCGA PanCancer studies. Abbreviations: STAD, stomach adenocarcinoma; ESCA, esophageal adenocarcinoma; HNSC, head and neck carcinoma; COAD, colorectal adenocarcinoma; LIHC, liver hepatocellular carcinoma; PAAD, pancreatic adenocarcinoma; PRAD, prostate adenocarcinoma; BRCA, breast invasive carcinoma; OV, ovarian serous adenocarcinoma; UCEC, uterine corpus endometrial carcinoma. **(B)** The mechanism of XRCC1-mediated maintenance of genomic stability. (i) Under normal cellular physiological conditions of BER-mediated DNA repair, oxidative or alkylating DNA-base damage is recognized by a DNA glycosylase, and the resulting repair intermediate is subsequently processed by APE1, Polβ, and DNA ligase III, coordinated by XRCC1 (55, 56). XRCC1 binding to the gap DNA promotes the timely release of PARP1 from DNA and prevents DNA–PARP1-complex-mediated genotoxicity (38). (ii) XRCC1-deficient cancer cells show increased accumulation of SSBs (5, 16), while PARP1 retention promotes the formation of trapped DNA–PARP1 complexes. (iii) Treatment of XRCC1-deficient cells with ATMi exacerbates replication-associated DNA damage during S-phase (37) and promotes genomic instability, including micronucleus formation and cytosolic DNA accumulation (36). All images and illustrations were generated using BioRender.

## Compelling questions

Dysregulation of DNA repair is a hallmark of many cancer types and contributes to genomic instability and tumor progression. BER serves as a major mechanism for repairing oxidative and alkylation-induced base lesions, thereby maintaining genomic integrity and cellular homeostasis. Several DNA repair factors, including XRCC1, are involved in processing single-base lesions. However, normal physiological DNA repair pathways can be compromised by various endogenous and exogenous factors (**Figure 1B**).

Collectively, this perspective emphasizes the critical role of XRCC1 in coordinating the interplay between genomic integrity and innate immune signaling in cancer cells. These findings also identify XRCC1 deficiency as a promising therapeutic vulnerability that could be strategically exploited to enhance antitumor immunity and advance the development of next-generation immunotherapeutic approaches for gastric cancer.

The proposed therapeutic framework comprises two mechanistic steps. (i) Inhibiting the DNA-damage response in XRCC1-deficient cancer cells leads to mitotic failure and accumulation of cytosolic DNA. Cancer cells with DNA repair deficiency are prone to accumulating high levels of DNA damage, and they are more dependent on DNA damage response (DDR) for survival than normal cells (25). Reactive oxygen species (ROS) are normal byproducts of numerous cellular processes, such as mitochondrial metabolism (26). At moderately increased levels, ROS induce DNA damage and promote genomic instability in cancer cells (27). To prevent genome instability, cancer cells rely on various DNA repair and DDR mechanisms (28–31). DNA damage is removed by DNA repair pathways, and DDR provides temporal and spatial coordination for DNA repair by controlling cell-cycle progression (32). Ataxia-Telangiectasia Mutated (ATM) is a serine/threonine kinase that plays a central role in response to DNA double-strand breaks (DSBs) (33) and is perhaps the most commonly altered DDR kinase across tumor types. Under normal conditions, ATM is recruited to DSBs, activated by autophosphorylation, and then initiates a cascade of phosphorylation events to promote DNA repair, cell-cycle arrest, and apoptosis. Tumor ATM deficiency has been associated with genomic instability and increased sensitivity to DNA-damaging agents in preclinical models and in some clinical contexts (34). The biological significance of XRCC1 loss is reflected in the accumulation of cellular poly(ADP-ribose) following DNA base damage, which disrupts the interactions among Polβ, XRCC1, and PARP1 (35). Therefore, the cooccurrence of XRCC1 loss and ATM dysfunction increases the incidence of genomic instability (36, 37). Previous studies have shown that XRCC1 is required to remove PARP1 from DNA, and its absence causes accumulation of PARP1-DNA complexes that can lead to genotoxicity (38). The recent study by Shahi et al. showed that pharmacological ATM inhibition exacerbates replication-associated double-strand breaks and leads to mitotic defects in XRCC1-deficient cancer cells (36) (**Figure 1B**). Micronucleus formation occurs in the context of cell division and is associated with defective metaphase centromere-kinetochore assembly or defects in anaphase (39, 40). Furthermore, mitotic defects in XRCC1-deficient cancer cells result in accumulation of DNA in the cytosolic compartment. This cytoplasmic DNA originated from both the nucleus and the mitochondria (36). (ii) Does XRCC1 deficiency promote the concurrent activation of innate immune signaling and programmed death-ligand 1 (PD-L1) upregulation? XRCC1-deficient cancer cells accumulate oxidative DNA-base damage products (41) and micronuclei (36) that serve as potential sources of cytoplasmic DNA. Oxidation of DNA bases does not impair or enhance the DNA’s ability to activate the cytosolic DNA sensor cyclic GMP-AMP synthase (cGAS), but the oxidized DNA is more resistant to cellular nucleases, which leads to greater induction of interferons (42). Cytoplasmic DNA (cytoDNA) species are emerging as key mediators of inflammation in diverse physiological and pathological contexts. Although cGAS-stimulator of interferon genes (STING) activation is essential for host defense and immune surveillance, inappropriate or sustained activation of this pathway can drive chronic inflammation, autoimmunity, and disease-associated immune dysfunction, thereby promoting tumor development and progression. Effective immunity therefore depends on tight regulatory control mechanisms that restrain cGAS-STING activity under homeostatic conditions while preserving the capacity for rapid and robust responses to diverse danger signals. Such balanced regulation is critical for maintaining immune homeostasis and preventing pathological consequences associated with aberrant pathway activation. The study by Shahi et al. (36) showed that loss of XRCC1 results in colocalization of cGAS with micronuclei, indicating the presence of cytoplasmic DNA. In addition, pharmacological inhibition of ATM significantly increased cytosolic DNA accumulation, which may activate cytosolic DNA sensors, including cGAS (43). Within cancer cells, the cGAS-STING pathway can be activated by cytosolic dsDNA originating from micronuclei, particularly in XRCC1-deficient cancer cells treated with ATM inhibitor (ATMi) (36). It is well documented that cytosolic dsDNA activates cGAS in tumor cells, leading to Cyclic guanosine monophosphate–adenosine monophosphate (cGAMP) production (44, 45). Accumulation of cGAMP in the cytoplasm activates the endoplasmic reticulum (ER)-resident receptor STING. Activation of STING is associated with phosphorylation of serine 365 (S365) within its C-terminal tail, triggering its translocation from the ER to the Golgi apparatus and promoting the recruitment of TANK-binding kinase 1 (TBK1) and interferon regulatory factor 3 (IRF3) (46, 47). Subsequent phosphorylation and activation of these signaling components induce the expression of type I interferons, particularly interferon-β (IFN-β). Type I IFN signaling enhances the activation of antigen-presenting cells (APCs), thereby promoting the cross-presentation of tumor antigens to CD8+ T cells and strengthening antitumor immune responses (48–50). Whereas acute cGAS-STING activation can support antitumor T-cell priming, chronic low-level activation in established tumors can rewire downstream signaling (51). In chromosomally unstable tumors, sustained cGAS-STING signaling preferentially activates noncanonical NF-κB and STAT3 pathways while suppressing IFN responses (51, 52). This dysregulated output supports tumor metastasis, angiogenesis, and immune suppression, effectively converting a tumor-suppressive mechanism into a tumor-promoting one (52, 53). The study by Shahi et al. shows that pharmacological inhibition of ATM in XRCC1-deficient cancer cells contributes to mitotic failure and cytosolic DNA accumulation (**Figure 2A**). Moreover, in the proposed model, XRCC1-proficient cancer cells are depicted as having relatively lower cancer-cell-intrinsic innate immune signaling, which may facilitate immune evasion. In contrast, XRCC1-deficient gastric cancer cells exhibit genomic instability and enhanced innate immune signaling that is further amplified by ATM inhibition (36) (**Figure 2B**). Additionally, ATMi-treated XRCC1-deficient cancer cells exhibit increased expression of PD-L1, which may contribute to adaptive immune resistance. These findings suggest that the genomic instability of cancer cells under DNA repair deficiency creates opportunities to enhance tumor immunogenicity, which may be leveraged to improve the efficacy of immunotherapies as a future therapeutic strategy.

**Figure 2:**
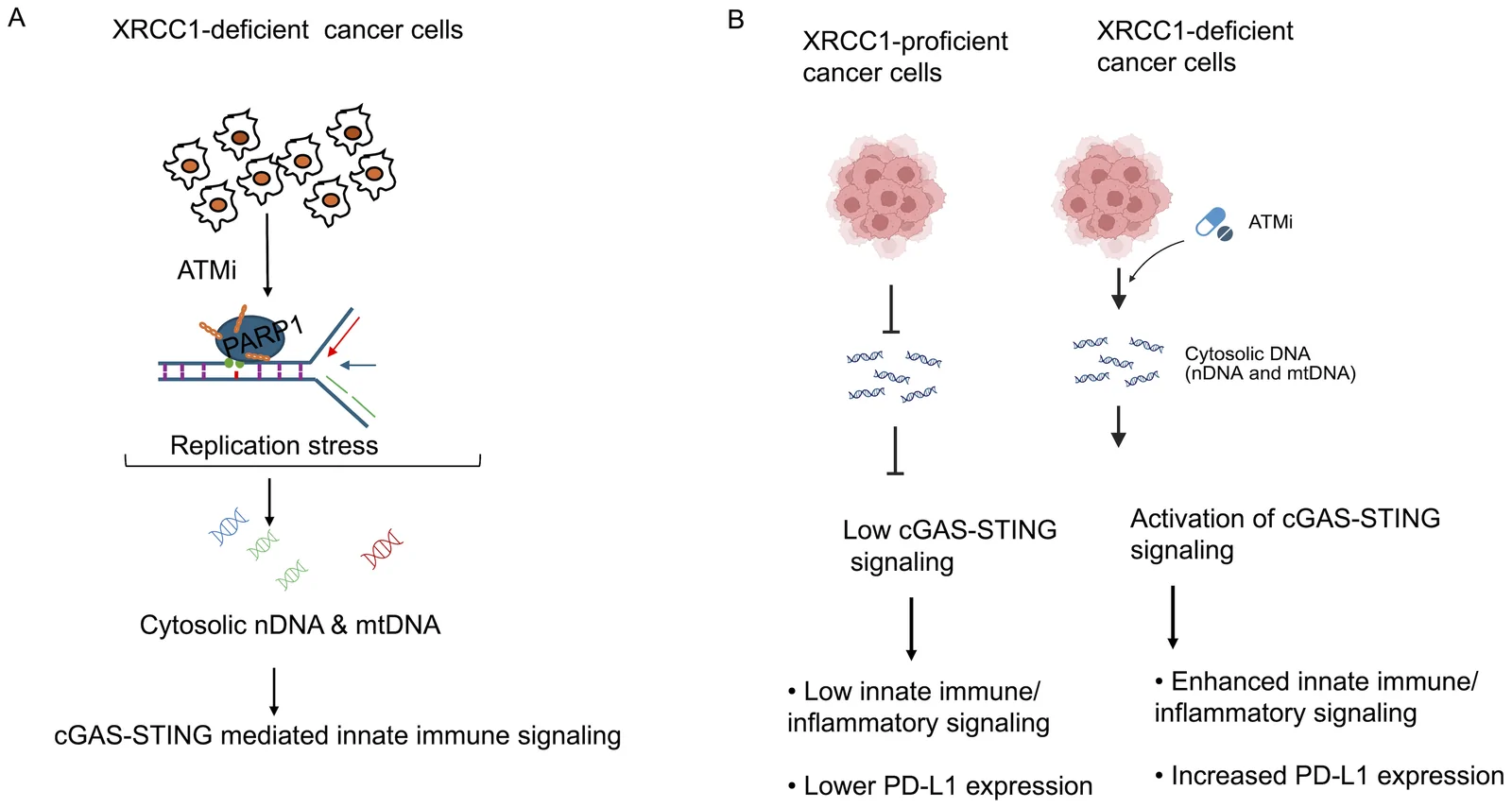
Inhibition of ATM enhances replication-associated DNA damage in XRCC1-deficient gastric cancer (GC) cells and triggers cGAS-STING-mediated inflammatory signaling. **(A)** ATM inhibition exacerbates replication-associated DNA damage in XRCC1-deficient cells, promoting genomic instability and cytosolic DNA accumulation (36, 37). **(B)** Proposed relationship between XRCC1 status, cancer-cell-intrinsic cGAS-STING signaling, and PD-L1 expression in the gastric cancer models. Schematic representation of the relationship between XRCC1-proficient and XRCC1-deficient cancer cells and innate immune signaling. XRCC1-proficient cancer cells exhibit low innate immune signaling and low levels of PD-L1 expression (right). In contrast, XRCC1-deficient cancer cells exhibit activated cGAS-STING-mediated innate immune signaling and increased PD-L1 expression (left). All images and illustrations were generated using BioRender.

## Future directions

Loss of XRCC1 in gastric cancer may provide an opportunity to lower the threshold for activation of innate immune signaling. Therefore, pharmacological intervention using DNA-damage response inhibitors, including ATM inhibitors, may maximize cGAS-STING-mediated innate immune signaling and enhance the induction of PD-L1 expression, which may represent an adaptive immune resistance mechanism. The cGAS-STING pathway is a critical cytosolic DNA-sensing system that bridges innate and adaptive immunity. Upregulation of PD-L1 in cancer may reflect an adaptive immunosuppressive response to pathway activation. In genomically unstable tumors, sustained inflammatory signals may contribute to remodeling of the tumor microenvironment toward immunosuppression (54). Harnessing the low expression of XRCC1 in cancer cells may provide a unique opportunity to identify therapeutic vulnerabilities that can be explored in future studies to enhance immune-based therapeutic responses. It is important to recognize that exploiting innate immune signaling associated with DNA repair-deficient cancer cells is only one factor in modulating the tumor microenvironment. It is also critical to distinguish cGAS-STING signaling intrinsic to XRCC1-deficient cancer cells from cGAS-STING signaling in immune cells such as antigen-presenting cells. Consideration of cGAS-STING signaling in stromal and immune cells may also be critical for defining the overall immunophenotype of the tumor microenvironment. This integrated understanding may help inform the development and application of immune checkpoint blockade therapies, potentially enhancing treatment responses as well as improving clinical outcomes for patients. It is important to note that reduced XRCC1 mRNA expression may be most informative as part of a broader biomarker signature rather than as a standalone predictor of treatment response. The clinical significance of XRCC1 deficiency may be strengthened when assessed in conjunction with other DNA repair and DNA damage response proteins. Moreover, XRCC1 protein expression and activity can be regulated through multiple mechanisms, including promoter methylation, posttranslational modifications, and functional genetic variants, each of which may influence DNA repair capacity and associated biological phenotypes. Future research must prioritize a combinatorial biomarker approach to integrate XRCC1 and other key DNA repair pathway components to provide a more effective framework for predicting therapeutic responses and identifying novel treatment opportunities in gastric cancer. Additionally, future studies combining different doses and schedules of ATMi with immune checkpoint blockade therapies are necessary to evaluate whether this combination yields additive or synergistic therapeutic benefits in XRCC1-deficient tumors. Specifically, different doses and schedules of ATM inhibitor treatment may influence cGAS-STING pathway activation, immune cell recruitment, and antitumor immune responses, thereby affecting the therapeutic efficacy of combination treatment strategies. A better understanding of the response thresholds that distinguish tumor-promoting signaling from effective antitumor immune responses is essential for optimizing therapeutic strategies and facilitating clinical translation.

## Data Availability

The original contributions presented in the study are included in the article/supplementary material. Further inquiries can be directed to the corresponding author.
